# IgG4:IgG RNA ratio differentiates active disease from remission in granulomatosis with polyangiitis: a new disease activity marker? A cross-sectional and longitudinal study

**DOI:** 10.1186/s13075-018-1806-6

**Published:** 2019-01-31

**Authors:** A. Al-Soudi, M. E. Doorenspleet, R. E. Esveldt, L. T. Burgemeister, A. E. Hak, B. J. H. van den Born, S. W. Tas, R. F. van Vollenhoven, P. L. Klarenbeek, N. de Vries

**Affiliations:** 1 Department of Rheumatology & Clinical Immunology and Amsterdam Rheumatology and Immunology Center (ARC), Amsterdam UMC, University of Amsterdam, Amsterdam, The Netherlands; 2 Department of Genome Analysis, Amsterdam UMC, University of Amsterdam, Amsterdam, The Netherlands; 3 Department of Experimental Immunology, Amsterdam Infection & Immunity Institute, Amsterdam UMC, University of Amsterdam, Amsterdam, The Netherlands; 4 Department of Vascular Medicine, Amsterdam UMC, University of Amsterdam, Amsterdam, The Netherlands

**Keywords:** Granulomatosis with polyangiitis, Biomarkers, Immunoglobulin G4, Disease activity, Quantitative polymerase chain reaction

## Abstract

**Objectives:**

An important limitation in granulomatosis with polyangiitis (GPA) is the lack of disease activity markers. Immunoglobulin G4-positive (IgG4^+^) B cells and plasma cells are implicated in the pathogenesis of GPA. We hypothesized that the presence of these cells in peripheral blood could serve as disease activity parameter in GPA.

**Methods:**

We included 35 proteinase 3-antineutrophil cytoplasmic antibodies-positive patients with GPA in a cross-sectional study. Active disease was defined as Birmingham Vasculitis Activity Score (BVAS) ≥ 3 (*n* = 15), remission as BVAS of 0 (*n* = 17), and low disease activity (LDA) as BVAS of 1–2 and clinical remission (*n* = 3). Healthy subjects (*n* = 10), patients with systemic lupus erythematosus (*n* = 24), and patients with rheumatoid arthritis (*n* = 19) functioned as control subjects. An additional longitudinal study was performed in ten patients with GPA. Using a validated qPCR test, we measured the IgG4:IgG RNA ratio in all groups and compared the results with known biomarkers.

**Results:**

The median qPCR score was higher in active GPA (21.4; IQR 12.1–29.6) than in remission/LDA (3.3; IQR 1.6–5.6) (Mann-Whitney *U* test, *p* < 0.0001) and outperformed other known disease activity parameters in detecting activity. A cutoff qPCR score of 11.2% differentiated active disease from remission/LDA accurately (AUC 0.993). The qPCR test correlated well with the BVAS (Spearman *r* = 0.77, *p* < 0.0001). In the longitudinal study, a decrease in BVAS correlated with qPCR score reduction (paired *t* test, *p* < 0.05).

**Conclusions:**

The IgG4:IgG RNA ratio in GPA accurately distinguishes active disease from remission and correlates well with disease activity in these single-center studies. If these results are confirmed in larger longitudinal studies, this test might help to steer treatment decisions in patients with GPA.

**Electronic supplementary material:**

The online version of this article (10.1186/s13075-018-1806-6) contains supplementary material, which is available to authorized users.

## Background

Granulomatosis with polyangiitis (GPA), formerly called Wegener’s disease, is a hallmark form of primary small vessel vasculitis. In GPA, the disease activity often fluctuates over time, making titration of therapy challenging [1]. Both disease activity and immunosuppressive therapies cause morbidity and mortality. Although therapeutic options have improved considerably, mortality is still high (15–25% mortality in the first 5 years after diagnosis) [2, 3]. In order to optimize treatment and improve survival, there is a pressing need for reliable biomarkers that reflect disease activity and can help to steer titration of therapy.

Current practice relies on general and unspecific inflammation parameters, such as erythrocyte sedimentation rate (ESR) and C-reactive protein (CRP), that do not correlate well with disease activity in vasculitis. Although GPA is associated with antineutrophil cytoplasmic antibodies (ANCA), these antibodies are more reliable in diagnosing the disease than in monitoring disease activity [4–9]. Instruments measuring disease activity, such as the Birmingham Vasculitis Activity Score (BVAS), have provided a solution and have been validated [10, 11]. However, they require that a clinician is convinced that symptoms scored are attributable to vasculitis activity and not to clinical mimics such as infections. This is often challenging in daily clinical practice.

A potential marker might be the presence of immunoglobulin G subtype 4 (IgG4)-positive B cells in peripheral blood. IgG4 itself and IgG4-positive (IgG4^+^) cells have been implicated in the pathogenesis of GPA. Several studies have reported increased levels of IgG4 antibodies in both serum and tissue in ANCA-associated vasculitis (AAV) [12–15]. IgG4^+^ B cells were also detected in inflamed tissue [13, 14, 16, 17].

In IgG4-related disease (IgG4-RD), a multiorgan disease known for high IgG4^+^ plasma cell infiltration in tissue, we recently demonstrated that the presence of IgG4^+^ B-cell receptor (BCR) clones in blood can serve as a diagnostic and disease activity biomarker [18]. These clones can be identified not only by next-generation sequencing (NGS) of the BCR repertoire but also by a simplified protocol using a qPCR test. The qPCR test accurately detected the IgG4^+^ BCR clones by measuring the RNA ratio between IgG4^+^ BCR and total IgG^+^ BCR [19].

Given the presence of IgG4^+^ B-cells in AAV, we hypothesized that the marker mentioned might also be informative in vasculitis as a disease activity marker. To this end, we tested the qPCR test in a cross-sectional and longitudinal cohort of patients with GPA.

## Materials and methods

### Study subjects

Patients meeting the American College of Rheumatology criteria and the Chapel Hill Consensus Conference definition for GPA were included in the cross-sectional study. In addition to these criteria, elevation in proteinase 3 (PR3)-ANCA had to be present at one point in the patient’s history. The diagnosis and these criteria were confirmed by two independent rheumatologists. Patients with concomitant other inflammatory diseases were excluded.

Patients were defined as having active disease if the BVAS version 3 showed a score of 3 points or higher [20]. Patients with a score of 0 were defined as being in remission. Patients with a BVAS of 1–2 who did not require ongoing therapy to be altered were defined as having low disease activity (LDA). The BVAS results were confirmed by two BVAS-certified clinicians. Both clinicians were blinded for the qPCR result, which was scored independently, and they subsequently reached agreement on the final BVAS. Limited disease, in contrast to systemic disease, was in agreement with present literature and was defined as the presence of manifestations of GPA that posed no immediate threat to either the patient’s life or the function of a vital organ [21]. In addition, activity parameters such as ESR (cutoff 30 mm/h), CRP (cutoff 5 mg/L), PR3-ANCA level (cutoff 7 kIU/L), and physician visual analogue scale (VAS) were determined. Age, sex, medication use, and the number of years since diagnosis were annotated.

Ten patients (seven with active disease) from the cross-sectional cohort were resampled at a later time point in the same manner, thus allowing longitudinal follow-up. One of these patients was resampled twice (Fig. 3, patient A).

As control groups, we included healthy control subjects (HC) and patients diagnosed with other autoimmune diseases with important B-cell involvement: systemic lupus erythematosus (SLE) and rheumatoid arthritis (RA). In addition, individuals who were age-matched to the active GPA cohort were included from our IgG4-RD cohort and used as positive controls [19]. In the SLE and RA groups, disease activity was determined according to current clinical standard using the Systemic Lupus Erythematosus Disease Activity Index (> 3 points) and Disease Activity Score in 28 joints (> 2.6 points) scores, respectively. The IgG4-RD control subjects had untreated disease and had active disease at the time of inclusion. Enrollment of all patients took place in the Academic Medical Center in Amsterdam, the Netherlands.

### Peripheral blood sampling and preparation

Peripheral blood was collected in PAXGene blood RNA tubes (catalog 762165; PreAnalytiX, Breda, The Netherlands) according to manufacturer’s instructions. RNA isolation was performed according to the manufacturer’s instructions using RNase-free DNase (Qiagen, Hilden, Germany) in the process. Complementary DNA was synthesized from 1000 ng of total RNA input using SuperScript III Reverse Transcriptase (Life Technologies, Carlsbad, CA, USA).

### qPCR

For a detailed description of the qPCR test used in this study, see an earlier publication by our group [19] and Additional file 1: Supplementary methods. Briefly, qPCR uses a common forward primer that binds upstream on the constant region of the BCR heavy chain and two specific reverse primers that bind downstream on the constant region of the BCR heavy chain. One reverse primer binds all possible IgG subtypes, and the other reverse primer is specific for the IgG4 subtype only. Subsequently, the IgG4:IgG ratio can be calculated from the qPCR results.

### Data analysis

qPCR data were analyzed using a LightCycler 480 instrument (Roche Diagnostics, Indianapolis, IN, USA) and LinRegPCR [22]. The analysis was always done by two blinded scientists separately. Statistical analyses were performed using Prism (GraphPad Software, La Jolla, CA, USA) and SPSS (IBM, Armonk, NY, USA) software. (Paired) *t* tests and analysis of variance (ANOVA) were used for normal distributions. Comparison of nonnormal distributed data was performed using the Mann-Whitney *U* test or Kruskal-Wallis test. Corrections for multiple testing were performed according to the Bonferroni or Dunn method. Similarly, correlation tests were performed using the Pearson method in case of normal distribution and the Spearman method in case of nonnormal distribution of data.

## Results

### Patient inclusion and characteristics

Thirty-five PR3-ANCA-positive patients with GPA were included. The median age was 56 years. Fifty-four percent of patients were female. The cohort was divided into an active disease group and a remission group on the basis of BVAS. A BVAS of 3 points or higher was regarded as active disease (15 patients, 43%). Seventeen patients (49%) had a BVAS of 0 (remission). Three patients (9%) had a BVAS of 1–2 (LDA). Patients in the LDA group had mild myalgia, arthralgia, and transient bloody nasal discharge that did not require change of treatment. The remission and LDA groups were combined for analysis (remission/LDA group, 20 patients [57%]). Sex, age, and the number of years since diagnosis did not differ significantly between both groups (Table 1). The symptoms scored in the BVAS per active disease and LDA subject are shown in Additional file 1: Table S1.

**Table 1:** Patient characteristics and disease activity parameters in total granulomatosis with polyangiitis cohort, active patients as determined by Birmingham Vasculitis Activity Score ≥ 3, and patients in remission/low disease activity as determined by Birmingham Vasculitis Activity Score < 3

	Overall	Active	Remission/LDA	*p* Value
Patient characteristics				
No. of patients	35	15	20	N/A
Sex (males/females)	16/19	7/8	9/11	ns
Age, years (mean + range)	54 (20–78)	59 (20–73)	50 (28–78)	ns
Years since diagnosis (median + IQR)	5 (1–12)	3 (1–12)	7 (1.3–12.8)	ns
Disease activity parameters				
BVAS (median + IQR)		8 (5–10)	0 (0–0)	< 0.0001
Physician VAS (median + IQR)		6 (2–8)	0 (0–0.5)	< 0.0001
CRP (mg/L) (median + IQR)		30.9 (6.5–70.7)	2.5 (1.9–8.7)	< 0.01
ESR (mm/h) (median + IQR)		20.5 (5–38)	5 (5–10)	< 0.05
Prednisolone (mg/d) (median + IQR)		7.5 (5–15)	5.8 (0.3–13.8)	ns
PR3-ANCA (kIU/L) (median + IQR)		124.6 (4.2–409)	16 (2.4–80.8)	ns

*Abbreviations: ANCA* Antineutrophil cytoplasmic antibodies, *BVAS* Birmingham Vasculitis Activity Score, *CRP* C-reactive protein, *ESR* Erythrocyte sedimentation rate, *LDA* Low disease activity, *ns* Not significant, *PR3* Proteinase 3, *VAS* Visual analogue scale

The active disease group had a higher BVAS than the remission/LDA group (median 8 points [IQR 5–10] vs 0 [0–0]; *p* < 0.0001). Other indicators of disease activity (median [IQR]) were significantly higher in the active group, such as physician VAS (6 [2–8] vs 0 [0–0.5] points; *p* < 0.0001), CRP (30.9 [6.5–70.7] vs 2.5 [1.9–8.7] mg/L; *p* < 0.01), and ESR (20.5 [5–38] vs 5 [5–10] mm/h; *p* < 0.05). However, prednisolone use (7.5 [5–15] vs 5.8 [0.3–13.8] mg/d; *p* = 0.39) and PR3-ANCA titer (124.6 [4.2–409] vs 22 [2.4–80.8] kIU/L; *p* = 0.17) did not differ significantly between the groups.

### qPCR test distinguishes active disease from remission/LDA in GPA

We hypothesized that the qPCR test could be used as a disease activity parameter because IgG4^+^ B-cells are implicated in the pathogenesis of GPA. To this end, we compared the qPCR scores between patients in the active disease group (BVAS ≥ 3) and those in the remission/LDA group (BVAS 0–2). The BVAS symptoms can be found in Additional file 1: Table S1.

**Fig. 1:**
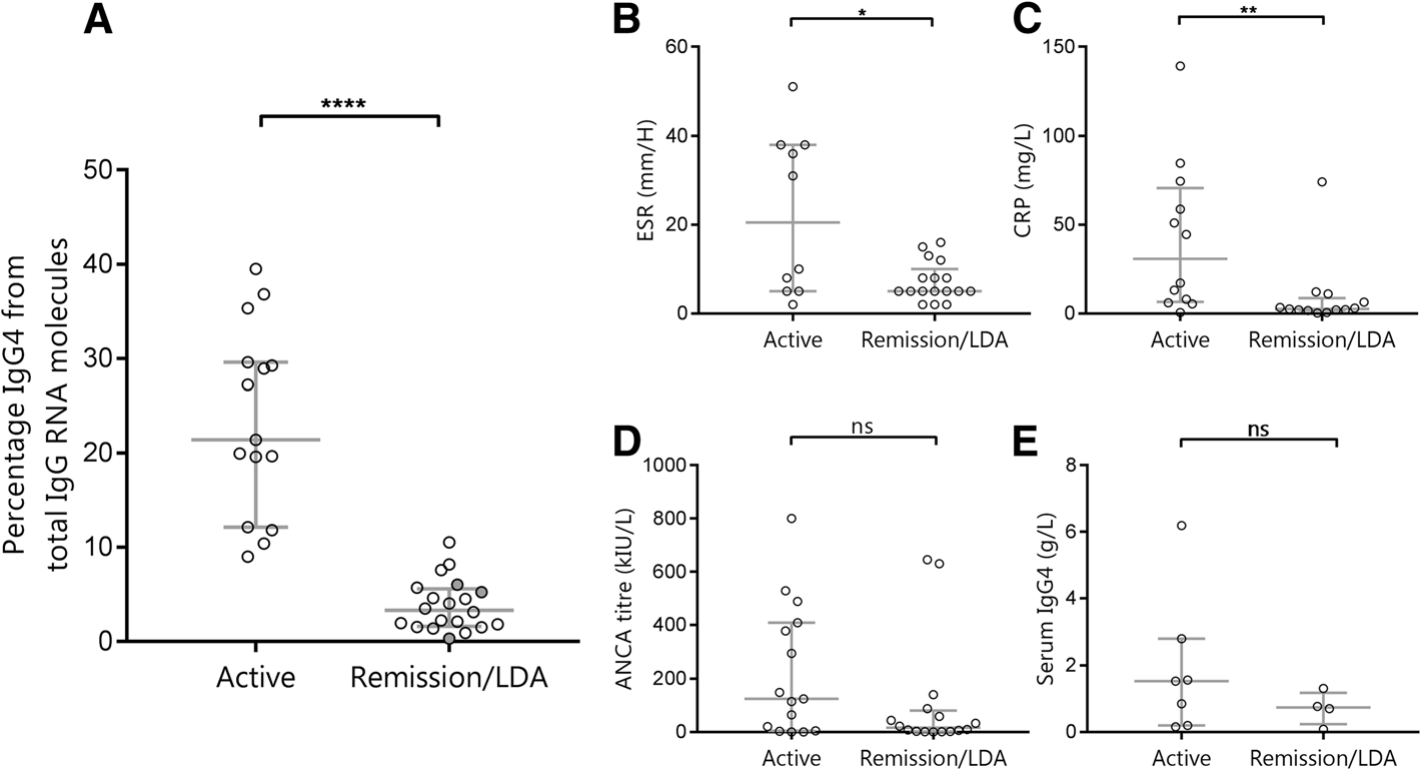
Disease activity parameters in active vs remission/low disease activity (LDA). **a** Scatter dot plot portraying the percentage of immunoglobulin G subtype 4 (IgG4) from total IgG RNA molecules in the active vs remission/LDA groups in patients with granulomatosis with polyangiitis (GPA). The gray dots within the remission/LDA group represent the three LDA patients. **b–e** Scatter dot plots showing erythrocyte sedimentation rate (ESR), C-reactive protein (CRP), antineutrophil cytoplasmic antibodies (ANCA), and serum IgG4, respectively, in the active vs remission/LDA groups in GPA (median with IQR). **** *p* < 0.0001, ** *p* < 0.01, * *p* < 0.05, ns = not significant

The median qPCR score was higher in the active disease group than in the remission/LDA group (median 21.4% [IQR 12.1–29.6] vs 3.3% [1.6–5.6]; *p* < 0.0001) (Fig. 1a). The ROC curve allowed accurate distinction between active disease and remission/LDA at a cutoff value of 11.2%, yielding a specificity of 100% and sensitivity of 87% (AUC 0.993) (Additional file 1: Table S2 and Additional file 2: Figure S1). In this case, specificity was preferred over sensitivity because of the expected clinical relevance (certainty of disease activity). When the LDA samples were excluded from the remission/LDA group, the results remained significant (Additional file 3: Figure S2).

To be of clinical relevance, the qPCR test has to outperform currently used disease activity parameters in GPA, such as ESR, CRP, and ANCA. To this end, the qPCR test was compared with the aforementioned markers using the BVAS as a gold standard for disease activity (Fig. 1b–e). We also tested serum IgG4 as a potential marker in patients when serum was available at the same time point.

The qPCR test outperformed CRP, ESR, and PR3-ANCA level in discriminating active disease from remission/LDA (Fig. 1, Table 2). CRP and ESR levels were also significantly different between the active disease group and the remission/LDA group, but lacked discriminatory capacity (Fig. 1b, c). Serum IgG4 and ANCA titers did not discriminate active disease from remission/LDA (Fig. 1d, e). The IgG4:IgG RNA ratio as determined by the qPCR test did not correlate with serum IgG4 (Additional file 4: Figure S3).

**Table 2:** Test characteristics of the different available tests detecting disease activity in our granulomatosis with polyangiitis cohort, using Birmingham Vasculitis Activity Score version 3 as gold standard, with Birmingham Vasculitis Activity Score ≥ 3 reflecting active disease

	ESR	CRP	ANCA titer	Physician VAS	qPCR test
Sensitivity (%)	50	92	73	80	86
Specificity (%)	100	69	38	90	100
Positive predictive value (%)	100	73	52	86	100
Negative predictive value (%)	77	90	60	86	91

*Abbreviations: ANCA* Antineutrophil cytoplasmic antibodies, *CRP* C-reactive protein, *ESR* Erythrocyte sedimentation rate, *VAS* Visual analogue scale

The cutoff values used were 30 mm/h for ESR, 5 mg/L for CRP, 7 kIU/L for ANCA, 2 or higher for physician VAS, and 11.2% for the qPCR test

Subsequently, the active disease group was further subdivided into a systemic disease group and a limited disease group (for definitions, *see* the “Materials and methods” section above). Both qPCR score and BVAS did not differ significantly between both groups (Additional file 5: Figure S4). Subdivision of the patients based on the most dominant organ with matching average qPCR score can be found in Additional file 1: Table S3.

Prednisolone use did not differ significantly between both groups (Table 1). More potent agents, such as rituximab (RTX) and cyclophosphamide (CYC) were used by 3 of 15 patients with active disease and by 1 patient in remission/LDA at the time of inclusion. Use of these agents did not result in lower qPCR scores in the active group (data not shown).

In summary, we demonstrated that the qPCR test distinguished active GPA from remission/LDA with very high accuracy using a cutoff at 11.2%. The predictive values of the qPCR test outperformed other frequently used laboratory parameters and the physician VAS score for disease activity.

### Elevated qPCR test scores observed in GPA are not observed in other autoantibody-associated diseases

**Fig. 2:**
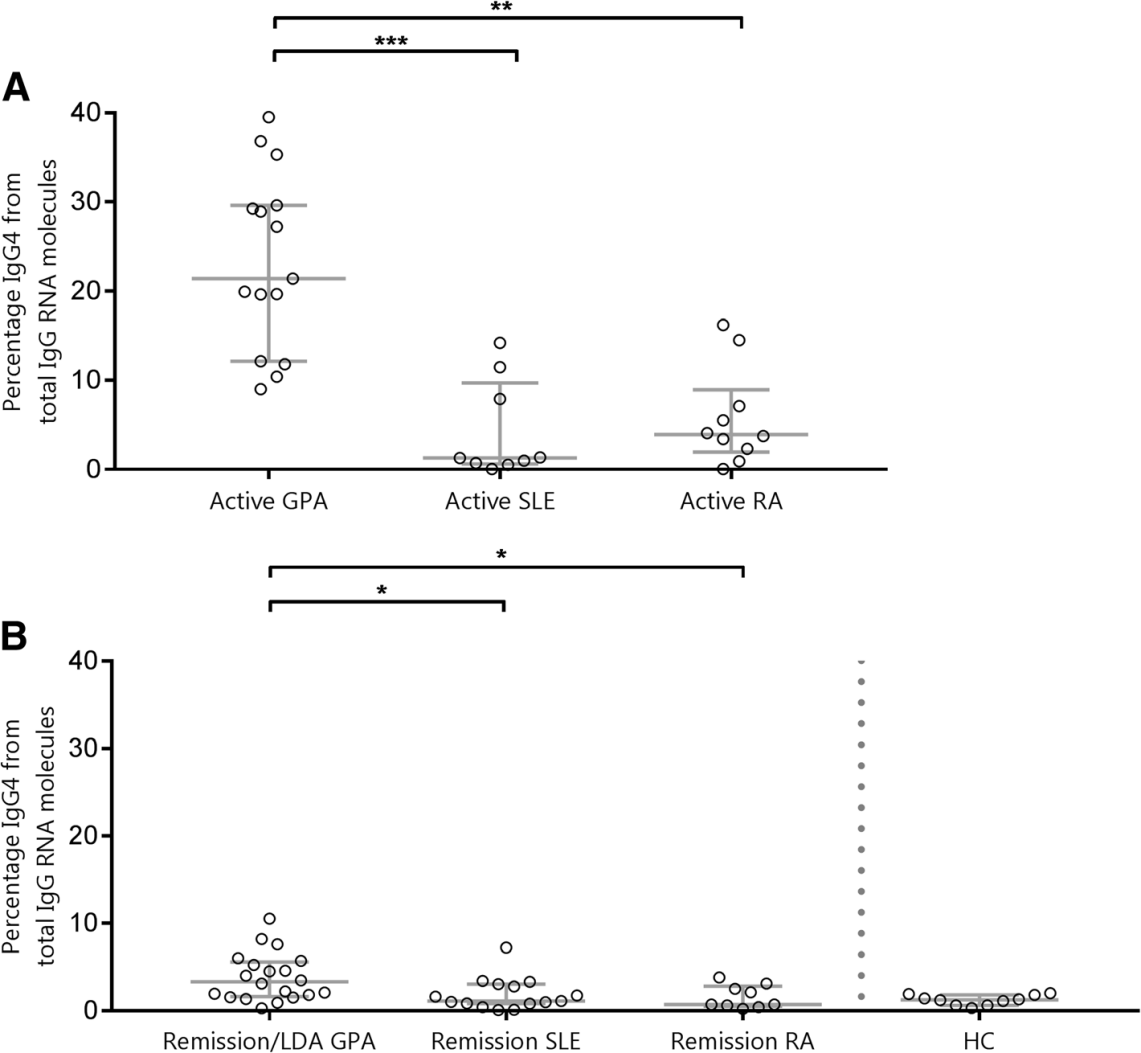
Granulomatosis with polyangiitis (GPA) vs disease control subjects and healthy control subjects (HC). Scatter dot plot portraying the percentage immunoglobulin G subtype 4 (IgG4) from total IgG RNA molecules in (**a**) active GPA, systemic lupus erythematosus (SLE), and rheumatoid arthritis (RA) and (**b**) GPA, SLE, and RA in remission/low disease activity (LDA) and HC. * *p* < 0.05, ** *p* < 0.01, *** *p* < 0.001 (Kruskal-Wallis test with Dunn’s correction)

These results prompted us to look in more detail into the specificity of these findings. We wondered whether the high qPCR could also be found in other autoantibody-associated diseases. To this end, we compared active GPA with active SLE and RA (Fig. 2). We added ten healthy control subjects as a negative reference group. We also made a comparison against 15 age-matched patients from the IgG4-RD cohort [19] with active disease as a positive reference. Details on the SLE, RA, and IgG4-RD cohorts can be found in Additional file 1: Table S4.

The qPCR score in the active GPA cohort was significantly higher than that in active disease control subjects (median 21.4% [IQR 12.1–29.6] vs 1.3% [0.6–9.7] in SLE [*p* < 0.001] and 3.9% [2–9] in RA [*p* < 0.01]) (Fig. 2a). The median qPCR score in the active GPA cohort was similar to that in the active IgG4 RD cohort (Additional file 6: Figure S5).

Interestingly, even when comparing scores of patients in remission/LDA, the qPCR scores were higher in GPA (median 3.8% [IQR 2–5.8] vs SLE 1.1% [0.8–3.1] [*p* < 0.05] and RA 0.7% [0.5–2.8] [*p* < 0.05]). The healthy control subjects had the lowest qPCR scores, and their result is shown as a reference value (Fig. 2b).

We observed that, collectively, the qPCR score was significantly higher in active GPA than in other active autoantibody-associated diseases. The same held true when we looked at the qPCR scores in patients with GPA in remission vs patients with control diseases in remission. This suggests that the strong upregulation of cellular IgG4 might be a phenomenon that is specific for GPA. Furthermore, this finding might contain important clues regarding the pathogenesis of GPA.

### qPCR test seems to be a promising monitoring tool in GPA

**Fig. 3:**
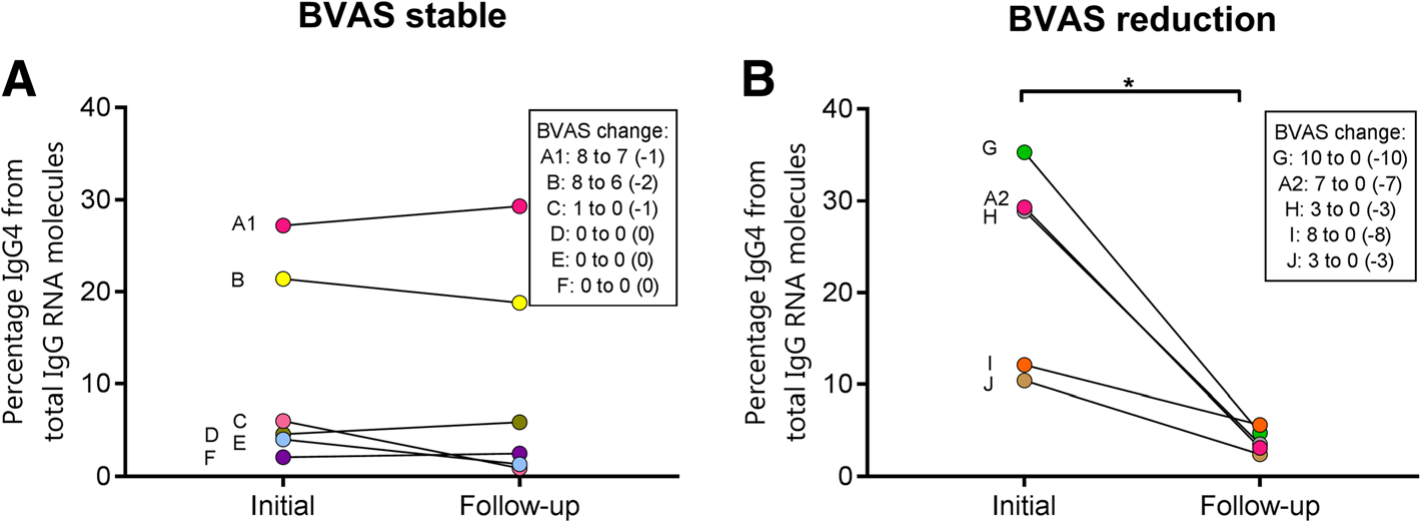
Longitudinal pilot. Longitudinal data in a scatter dot plot portraying the percentage of immunoglobulin G subtype 4 (IgG4) from total IgG RNA molecules in (**a**) patients who showed no or minor changes in Birmingham Vasculitis Activity Score (BVAS) and (**b**) patients who showed a decrease of 3 points or more in BVAS after follow-up

For a disease activity marker to be used in a clinical setting, the result should change when the disease activity changes and should stay stable when the disease activity is stable. To test whether this was true for the qPCR test, we repeated the qPCR test in ten patients at a second time point, creating a longitudinal cohort. Six patients were resampled while in steady state (BVAS change < 3 points). Four patients were in stable remission/LDA, and two (A1 and B) were unsuccessfully treated between the initial and follow-up time points (Fig. 3a). Five patients were sampled after a BVAS decrease of 3 or more points. Of interest, patient A is the same in both panels of Fig. 3. This patient was sampled three times: once after unsuccessful treatment (Fig. 3a) and once after successful treatment (Fig. 3b).

In the group with a stable BVAS, the qPCR scores did not change (Fig. 3b). We observed a significant decrease in qPCR ratio in the group of patients with a BVAS reduction > 3 points (paired *t* test, *p* < 0.05) (Fig. 3b).

### qPCR differentiates true vasculitis activity from mimics in six individuals

The unmet need for biomarkers in GPA lies in the ability to distinguish GPA activity from mimics such as infections. To investigate whether the IgG4 qPCR would be able to differentiate between GPA activity and mimics, we performed the IgG4 qPCR test in a real-life clinical situation where clinical symptoms could be caused by either GPA activity or GPA mimics. We tested six patients. Two patients were from the cross-sectional cohort (CS1 and CS2), and four patients were from the longitudinal cohort (E, A2, A1, B) (Table 3). We used additional diagnostic tests and clinical follow-up to determine whether the symptoms were caused by GPA activity or a mimic.

**Table 3:** Overview of six individuals tested while suspected of having granulomatosis with polyangiitis flare

Identifier	CS1	CS2	E	A2	A1	B
Cohort	CS	CS	L	L	L	L
Age, years	59	64	63	65	64	53
Dominant organ at presentation	CNS	Lungs	Lungs	Lungs	ENT	Eyes
ESR	13	NA	20	NA	38	2
CRP	NA	74.2	1.3	128.1	44.5	10.6
ANCA	1.3	NA	264	74.5	114.9	NA
Outcome	Radiculopathy	Community-acquired pneumonia	*Streptococcus pneumoniae* pneumonia	Influenza A pneumonia	GPA	GPA
BVAS (in case of GPA)					7	6
qPCR score	1.5%	0.9%	1.3%	3.3%	29.3%	18.8%

*Abbreviations: ANCA* Antineutrophil cytoplasmic antibodies, *BVAS* Birmingham Vasculitis Activity Score, *CNS* Central Nervous System, *CRP* C-reactive protein, *CS* Cross-sectional, *ESR* Erythrocyte sedimentation rate, *GPA* Granulomatosis with polyangiitis, *L* Longitudinal, *NA* Not annotated at visit

Two columns at right highlight the individuals in whom the suspicion turned out to be true. Identifiers for the CS patients were randomly assigned for this overview’s purposes. Identifiers for L patients match the identifiers in Fig. 3

In patients in whom the symptoms were caused by mimics, the qPCR scores were normal (Table 3, columns 2–5). In CS1, CS2, and A2, the CRP levels were elevated due to the ongoing infection. ANCA titer was elevated during infection in two cases (CS2, A2). The qPCR scores were very high in the patients in whom the symptoms were caused by GPA activity (A1, B), indicating that the test is a promising way to distinguish true vasculitis activity from (infectious) mimics. These results need to be confirmed in a larger and more diverse group of patients with GPA.

### Correlation between the qPCR test and disease activity as measured by BVAS

**Fig. 4:**
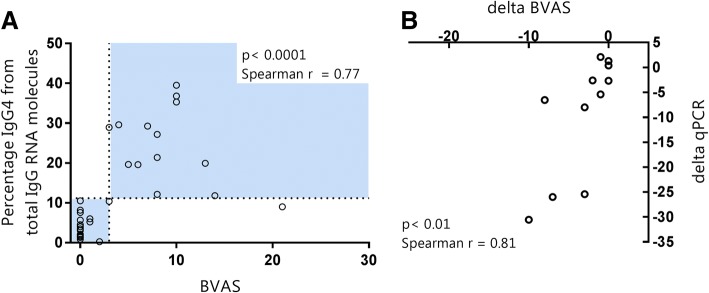
Correlation between qPCR test and Birmingham Vasculitis Activity Score (BVAS). **a** Correlation between BVAS (*x*-axis) and the percentage of immunoglobulin G subtype 4 (IgG4) from total IgG RNA molecules (*y*-axis) in all individuals. The blue parts of the graph depict the areas where patients are expected in case of remission/low disease activity (BVAS < 3 and qPCR < 11.2%, lower left quadrant) and activity (BVAS ≥ 3 and qPCR ≥ 11.2%, upper right quadrant). **b** Correlation between ∆BVAS and ∆qPCR in the ten longitudinal cohort subjects

The qPCR test accurately distinguished active disease from remission/LDA and decreased when BVAS decreased after treatment. To investigate the correlation in more detail, we analyzed the relationship between the BVAS and qPCR test (Fig. 4a).

We observed that the BVAS and qPCR showed a significant correlation (*p* < 0.0001, Spearman *r* = 0.77). In the ten patients from the longitudinal cohort, we calculated a ∆BVAS and ∆qPCR (Fig. 4b). Overall, these values correlated significantly (*p* < 0.01, Spearman *r* = 0.81). Correlation was excellent in the lower disease range. However, the correlation slightly decreased, especially in the patients showing high ∆BVAS and ∆qPCR. Further studies are needed to analyze whether this reflects nonlinearity when measuring vasculitis activity by the qPCR, by the BVAS, or both.

## Discussion

In these single-center studies, we demonstrated that the qPCR test associates with disease activity in GPA as measured by BVAS-V3 and outperforms current markers such as PR3-ANCA titer, CRP, ESR, and serum IgG4 level. We confirmed the association in a small longitudinal study. As such, we suggest the qPCR test as a potential new disease activity marker in GPA.

Over the last decade, the BVAS has been instrumental in creating uniformity in scoring GPA disease activity. However, an important limitation is the fact that the scoring system assumes that the physician is certain that the scored symptoms are caused by active vasculitis. When in doubt, physicians turn to available biomarkers such as ESR, CRP, ANCA titer, or sometimes even serum IgG4. In line with previous studies, we found that these markers have limitations in specificity (ANCA, CRP) and sensitivity (ESR) (Table 2) [4, 7–9]. To the best of our knowledge, no other more sensitive or more specific disease activity markers have been identified until now [4, 5]. Although our findings appear robust, our study is limited by its size and the limited amount of longitudinal data. The value of the qPCR test should be evaluated in more detail in further longitudinal studies in which there is special attention to disease activity mimics such as infections. Importantly, in four cases in this study, we demonstrated that the qPCR remained low in episodes of mimicked vasculitis activity (Table 3). This further suggests that the qPCR test might be a specific marker that can identify true vasculitis activity.

The qPCR test is based on earlier NGS results in IgG4-RD, where we showed that the qPCR test can accurately discriminate the biliary form of IgG4-RD from its mimics and quantitatively correlates with disease activity in the individual patient [19]. The qPCR test was validated against NGS as a gold standard and showed reproducible results in independent cohorts. We do want to point out that experience with the qPCR test is limited to our center.

The fact that similar results are now found in AAV is not unexpected, given the clear overlap in clinical, histological, and even laboratory features between IgG4-RD and GPA [13–15, 17, 23–31]. IgG4 might also play a role in other forms of (ANCA-associated) vasculitis. For example, IgG4 serum levels have been correlated with disease activity in eosinophilic GPA [12, 32]. Although there are also many differences in regard to disease presentation between vasculitis and IgG4-RD, the involvement of IgG4^+^ cells, as measured by the qPCR test in this study, raises the hypothesis that there is a shared (patho-)physiological mechanism involved. This mechanism might by exploited as a potential therapeutic target in the future.

In peripheral blood, the qPCR test quantifies RNA from both IgG4- and IgG-producing cells. The test result is subsequently calculated by dividing the amount of IgG4 RNA by that of total IgG RNA. Thus, an increase in this test result does *not* reflect an increase in the amount of IgG4 protein present in serum, but rather represents either an increase in the number of IgG4-producing B-lineage cells and/or an increase in the number of IgG4 mRNA molecules expressed per cell, such as during differentiation from mature B cells to plasmablasts. Although we extensively studied the relationship between the qPCR test and the presence of such BCR clones in IgG4-RD previously, we once more confirmed this in one of the patients with GPA (Additional file 7: Figure S6).

It is puzzling that the presence of IgG4^+^ B cells in the blood correlates with disease activity in GPA. We propose two hypotheses that may explain our findings. The first is that IgG4^+^ clones are pathogenic in GPA. This may seem unlikely owing to the theoretical inability of IgG4 to initiate pathogenic responses [33–35], but in diseases such as pemphigus and myasthenia gravis, IgG4 antibodies have been demonstrated to have pathogenic properties [18, 36–41]. The second hypothesis is that the IgG4 response represents a counterregulatory mechanism following vasculitis activity to dampen the inflammation. This would be more compatible with the theory of IgG4 being a suppressive immunomodulator [34]. The lack of correlation between protein IgG4 in serum and disease activity is probably a reflection of continued stable production of IgG4 by plasma cells located in bone marrow or other locations that is unaffected by disease activity elsewhere. We demonstrated that our IgG4/IgG mRNA ratio as measured by the qPCR test, which correlates with disease activity, reflects the real-time differentiation of IgG4-producing B cells to plasmablasts and migration of these cells through the blood to other locations, such as from site of inflammation to bone marrow [18, 19, 42]. It is conceivable that this process begins before the onset of disease activity, thus predicting the onset of a flare. This concept will need further exploration in longitudinal studies.

Subdivision of the active GPA group into limited (five individuals) and systemic (ten individuals) disease groups did not show any differences in qPCR score, suggesting that the IgG4^+^ B-cell upregulation is not linked to disease manifestation (Additional file 5: Figure S4). However, it has to be noted that these two sample groups were small and that further subdivision based on organ involvement was not possible, because sample groups would be too small to record any significant differences.

Furthermore, one could argue that although not significantly different between the groups, the remission/LDA group had a longer duration since diagnosis and therefore possibly had more cumulative treatment, which could explain these results. However, years since diagnosis and qPCR score did not correlate, indicating that a longer disease duration is unlikely to yield lower qPCR scores (Additional file 8: Figure S7). Instead, we believe that disease activity and shorter duration since diagnosis are linked because it has extensively been demonstrated that disease flares tend to occur within the first few years after GPA diagnosis.

Finally, there were several interesting observations with regard to immunosuppressive drugs in our cohort. Use of prednisone was similar in both the active and remission/LDA groups. Prednisone is a potent agent frequently used in induction of remission. However, only after reaching remission (while prednisone is often continued) did the qPCR scores decrease. Second, most patients were included at first presentation of active disease (de novo, flare), when more potent agents such as RTX and CYC are yet to be initiated. Only four subjects were included at a moment during induction therapy when they had received one or more cycles of RTX or CYC. Our findings do not indicate that the IgG4 qPCR score is directly altered by these agents, implying that the IgG4 qPCR reflects disease activity. Nevertheless, we want to emphasize that this study is too small to make definite conclusions. A larger longitudinal study is needed to study the relationship between drugs and IgG4 qPCR scores. Such a study would need enough responders and nonresponders receiving each drug to differentiate the effect of a drug from a change in disease activity as a cause of the change in IgG4:IgG mRNA ratio. Moreover, the long-term effects of B-cell-depleting therapies such as RTX on the IgG4:IgG mRNA ratio are unknown and would require a study that addresses the relationship between the IgG4 qPCR score and circulating B-cell subsets, including plasmablasts. In IgG4-RD, prednisone was also shown to decrease plasmablast counts, which in turn was correlated with disease activity [18, 19]. Dissecting these relationships might help elucidate the pathophysiology of IgG4^+^ cells and their responses in GPA.

## Conclusions

We report that the IgG4:IgG RNA ratio as measured by a qPCR test might be a new and promising disease activity marker in GPA. The qPCR test is able to accurately distinguish disease activity from remission/LDA and outperforms frequently used disease activity parameters in GPA. Furthermore, it seems feasible to use the qPCR test as a monitoring tool and discriminate disease activity from mimics such as infection. A major limitation of this study is the lack of independent validation of the results. Therefore, the value of the test should be further explored in longitudinal studies. If the present results are confirmed, it will help to optimize treatment, thus preventing morbidity and mortality from under- and overtreatment.

## Additional files

Additional file 1: Supplementary methods. **Table S1.** BVAS division per active (**a**) and low disease activity (LDA) subject with the most involved (dominant) organ and qPCR score. The final column shows the number the subject has in Fig. 3. **Table S2.** Coordinates of the curve: the critical cutoff values for the qPCR test, producing different values for sensitivity and specificity. **Table S3.** Average qPCR score per dominant organ. **Table S4.** Patient characteristics for SLE (*n* = 24) and RA (*n* = 19) cohorts. IgG4-RD (*n* = 15) and HC (*n* = 10) are shown as reference groups. na = not annotated, ns = not significant. (DOCX 25 kb)

Additional file 2: **Figure S1.** ROC curve portraying sensitivity and specificity of the qPCR test to distinguish active GPA from remission. The blue arrow points toward the cutoff that yields the highest specificity with the least loss of sensitivity. (JPG 40 kb)

Additional file 3: **Figure S2.** Active GPA vs remission GPA (without LDA). Scatter dot plot portraying the percentage of IgG4 from total IgG RNA molecules in the active vs remission groups in GPA without LDA. (BMP 3716 kb)

Additional file 4: **Figure S3.** Serum IgG4 vs qPCR test. Scatter dot plot portraying the correlation between serum IgG4 (*x*-axis) and the percentage of IgG4 from total IgG RNA molecules (*y*-axis) (Spearman’s *r* correlation). (BMP 3413 kb)

Additional file 5: **Figure S4.** Systemic GPA vs limited GPA. Scatter dot plot with in (**a**) the qPCR result within the active GPA group divided by systemic and limited disease and in (**b**) the matching BVAS for this group. (BMP 13494 kb)

Additional file 6: **Figure S5.** Active GPA vs active IgG4-RD. Scatter dot plot portraying the percentage of IgG4 from total IgG RNA molecules in the active GPA vs active IgG4-RD control groups. ns = not significant. (BMP 3861 kb)

Additional file 7: **Figure S6.** NGS data on IgG4^+^ BCR clones. NGS data portraying the frequency of IgG4^+^ BCR clones (red) and IgG clones (black) in a GPA patient (*x*-axis, left) and a representative healthy control subject (*x*-axis, right). See an earlier study for a complete description of the amplification protocol [43]. Briefly, a linear amplification is performed using six primers specific for the V segment of the BCR heavy chain. Each primer has a nonbinding common sequence on the 5′ end for the second amplification step. The primers cover all known V-segment alleles. After purification, amplification is performed using the common sequence introduced by the V primers and a common primer on the boundary of the J segment and C segment. Primers are available upon request. Further processing was performed according to the protocol for the MiSeq platform (Illumina, San Diego, CA, USA), which was used for NGS. NGS results were analyzed using a custom pipeline [44]. (PDF 60 kb)

Additional file 8: **Figure S7.** Years since diagnosis vs qPCR score. The *r* and *p* values demonstrate no correlation between disease duration and qPCR score. (BMP 3328 kb)

## Abbreviations

AAV: Antineutrophil cytoplasmic antibodies-associated vasculitis
ANCA: Antineutrophil cytoplasmic antibodies
BCR: B-cell receptor
BVAS: Birmingham Vasculitis Activity Score
CRP: C-reactive protein
CYC: Cyclophosphamide
ESR: Erythrocyte sedimentation rate
GPA: Granulomatosis with polyangiitis
IgG: Immunoglobulin G
IgG4: Immunoglobulin G subtype 4
IgG4-RD: Immunoglobulin G subtype 4-related disease
LDA: Low disease activity
NGS: Next-generation sequencing
PR3: Proteinase 3
RA: Rheumatoid arthritis
RTX: Rituximab
SLE: Systemic lupus erythematosus
VAS: Visual analogue scale
