# Supplementary material for: IgG4:IgG RNA ratio differentiates active disease from remission in granulomatosis with polyangiitis: a new disease activity marker? A cross-sectional and longitudinal study
Source: Arthritis Res Ther. 2019 Jan 31;21:43. doi: 10.1186/s13075-018-1806-6 (PMC6357433; doi:10.1186/s13075-018-1806-6)
Supplement: Supplementary file 1 — Supplementary methods. Table S1. BVAS division per active (a) and low disease activity (LDA) subject with the most involved (dominant) organ and qPCR score. The final column shows the number the subject has in Fig. 3. Table S2. Coordinates of the curve: the critical cutoff values for the qPCR test, producing different values for sensitivity and specificity. Table S3. Average qPCR score per dominant organ. Table S4. Patient characteristics for SLE (n = 24) and RA (n = 19) cohorts. IgG4-RD (n = 15) and HC (n = 10) are shown as reference groups. na = not annotated, ns = not significant. (DOCX 25 kb) [file 13075_2018_1806_MOESM1_ESM.docx]

Additional file 1

**Supplementary methods**

All RNA samples were masked and randomized prior to testing to limit any form of bias. Triplicate reactions were performed for the forward primer in combination with either of the reverse primers. Reactions were performed in 10μL total volume in the presence of 10pmol of the forward and reverse primers, cDNA from 50ng RNA input and 5μL SensiFAST SYBR Lo-ROX reagent (catalog #BIO-94005, Bioline, GC Biotech, Alphen a/d Rijn, Netherlands) for 40 cycles (95°C for 2 minutes, 40 cycles [95°C for 5 seconds; 60°C for 10 seconds; 72°C for 20 seconds], followed by a melting curve [95°C for 5 seconds; 65° C for 1 minute; 97° C continuous]), using the LightCycler480 system (Roche Diagnostics, Almere, Netherlands).

| **State** | **BVAS** | **Involvement** | **Dominant organ** | **qPCR score** | **Active longitudinal patient** |
| --- | --- | --- | --- | --- | --- |
| A | 8 | New arthralgia/arthritis (1), new fever (2), bloody nasal discharge (4), headache (1) | ENT | 27,2% | A |
| A | 8 | New arthralgia/arthritis (1), conjunctivitis (1), bloody nasal discharge (4) and paranasal sinus involvement (2) | ENT | 12,1% | I |
| A | 6 | New bloody nasal discharge, paranasal sinus involvement, conductive hearing loss (6 , max ENT) | ENT | 19,6% |  |
| A | 8 | New scleritis (2), bloody nasal discharge (4), paranasal sinus involvement (2) | ENT | 21,4% | B |
| A | 7 | New fever (2), headache (1), bloody nasal discharge (4) | ENT | 29,3% |  |
| A | 3 | Persistent Infiltrates lung (2), arthralgia/arthritis (1) | Lungs | 28,9% | H |
| A | 10 | New nasal discharge (4), conductive hearing loss (2), endobronchial involvement (4) | Lungs | 36,8% |  |
| A | 10 | New Significant proptosis, blurred vision (6, max of mucous membranes/eyes), bloody nasal discharge (4) | Mucous membranes/eyes | 35,3% | G |
| A | 3 | Persistent sensory peripheral neuropathy (3) | Nervous system | 10,4% | J |
| A | 4 | Persistent sensory peripheral neuropathy (3) persisting ulcus (1) | Nervous system | 29,6% |  |
| A | 21 | New artralgia/arthritis (1), cavities (3), haematuria (6), mononeuritis multiplex (9), scleritis (2) | Nervous system | 9,0% |  |
| A | 14 | New arthralgia/arthritis (1), proteinuria (4), haematuria (6) and nodules (3) | Renal | 11,8% |  |
| A | 5 | Persistent haematuria (3) and proteinuria (2) | Renal | 19,7% |  |
| A | 13 | New artralgia/arthritis (1), haematuria (6), infiltrate (4), nodules (2) | Renal | 19,9% |  |
| A | 10 | New fever (2), bloody nasal discharge (4), proteinuria (4) | Renal | 39,5% |  |
| LDA | 2 | Persistent bloody nasal discharge (2) |  | 0,3% |  |
| LDA | 1 | Persistent arthralgia (1) |  | 5,2% |  |
| LDA | 1 | Persistent myalgia (1) |  | 6,0% |  |

Table S1 BVAS division per active (A) and low disease activity (LDA) subject with the most involved (dominant) organ and qPCR score. The final column shows the number the subject has in figure 3.

| **qPCR score equal or greater than** | **Sensitivity** | **1 - Specificity** | |
| --- | --- | --- | --- |
| 10.45 | 86.7% | 95% | |
| 11.15 | 86.7% | 100% | |
| 11.97 | 80% | 100% | |
|  | | |  |

Table S2 Coordinates of the curve: The critical cut-off values for the qPCR test, producing different values for sensitivity and specificity.

| **Dominant organ** | **N patients** | **Average qPCR score** |
| --- | --- | --- |
| ENT | 5 | 21.9% |
| Lungs | 2 | 32.9% |
| Mucous membranes/eyes | 1 | 35.3% |
| Nervous system | 3 | 16.3% |
| Renal | 4 | 22.7% |

Table S3 Average qPCR score per dominant organ.

| **Patient Characteristics** | | | | | | | | | |
| --- | --- | --- | --- | --- | --- | --- | --- | --- | --- |
|  | **SLE active** | **SLE remission** |  | **RA active** | **RA remission** |  | **IgG4-RD active** | **HC** |  |
| N | 9 | 15 |  | 10 | 9 |  | 15 | 10 |  |
| Sex (male-female) | 0-9 | 2-13 |  | 1-9 | 0-9 |  | 14-1 | 6-4 |  |
| Age (mean + range) | 36 (20-57) | 41 (19-84) | *ns* | 51 (35-67) | 47 41-57) | *ns* | 64 (63-65) | 51 (48-62) |  |
| Years since diagnosis (median + IQR) | 6 (0.5-9.5) | 10 (4-13) | *ns* | na | na |  | 0 (0-0) |  |  |
| **Disease activity parameters** | | | | | | | | | |
|  | **SLE active** | **SLE remission** |  | **RA active** | **RA remission** |  | **IgG4-RD active** | **HC** |  |
| SLEDAI (median + IQR) | 4 (4-14.5) | 0 (0-0.25) | *<0.001* |  |  |  |  |  |  |
| DAS28 (median + IQR) |  |  |  | 3.2 (2.7-3.5) | 1.8 (1.3-2.4) | *<0.05* |  |  |  |
| CRP (mg/L) (median + IQR) | 28.5 (5-98) | 3 (1.5-4) | *<0.05* | 4 (2-33) | 1 (0.5-2) | *ns* |  |  |  |
| ESR (mm/H) (median + IQR) | 29 (13-51.5) | 8 (5-15) | *<0.01* | 8 (3.5-17.5) | 6 (5-8) | *ns* |  |  |  |
| Prednisolone (mg/day) (median + IQR) | 20 (0-35) | 0 (0-0) | *<0.01* | 0 (0-0) | 0 (0-3.8) | *ns* | 0 (0-0) |  |  |
| Biological use (%) | 0 | 0 | *ns* | 57 | 25 |  | 0 |  |  |
| Anti-double stranded DNA (median + IQR) | 176 (18-337) | 103 (33-514) | *ns* |  |  |  |  |  |  |
| Anti-citrullinated protein antibodies (%) |  |  |  | 100 | 100 |  |  |  |  |
| Rheumatoid Factor (%) |  |  |  | 100 | 100 |  |  |  |  |

Table S4 Patient characteristics for SLE (N=24) and RA (N=19) cohorts. IgG4-RD (N=15) and HC (N=10) are shown as reference groups. na= not annotated, ns= not significant
