# Supplementary figures and images for: IgG4:IgG RNA ratio differentiates active disease from remission in granulomatosis with polyangiitis: a new disease activity marker? A cross-sectional and longitudinal study
Source: Arthritis Res Ther. 2019 Jan 31;21:43. doi: 10.1186/s13075-018-1806-6 (PMC6357433; doi:10.1186/s13075-018-1806-6)

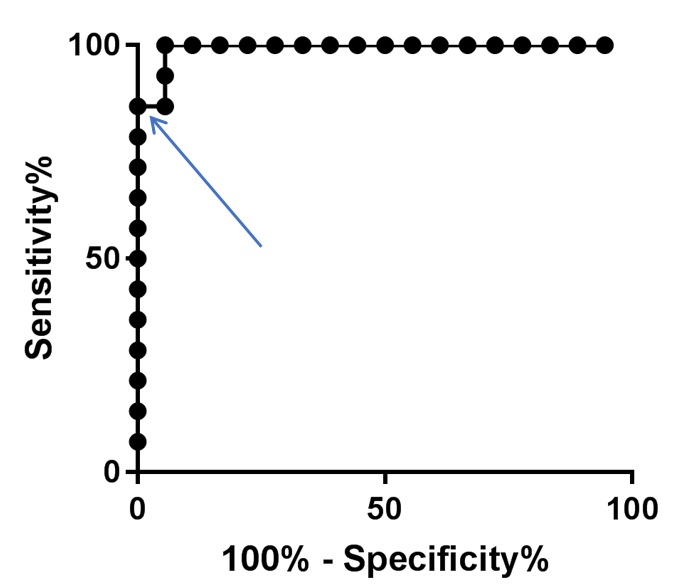

Supplement: Supplementary file 2 — Figure S1. ROC curve portraying sensitivity and specificity of the qPCR test to distinguish active GPA from remission. The blue arrow points toward the cutoff that yields the highest specificity with the least loss of sensitivity. (JPG 40 kb) [file 13075_2018_1806_MOESM2_ESM.jpg]

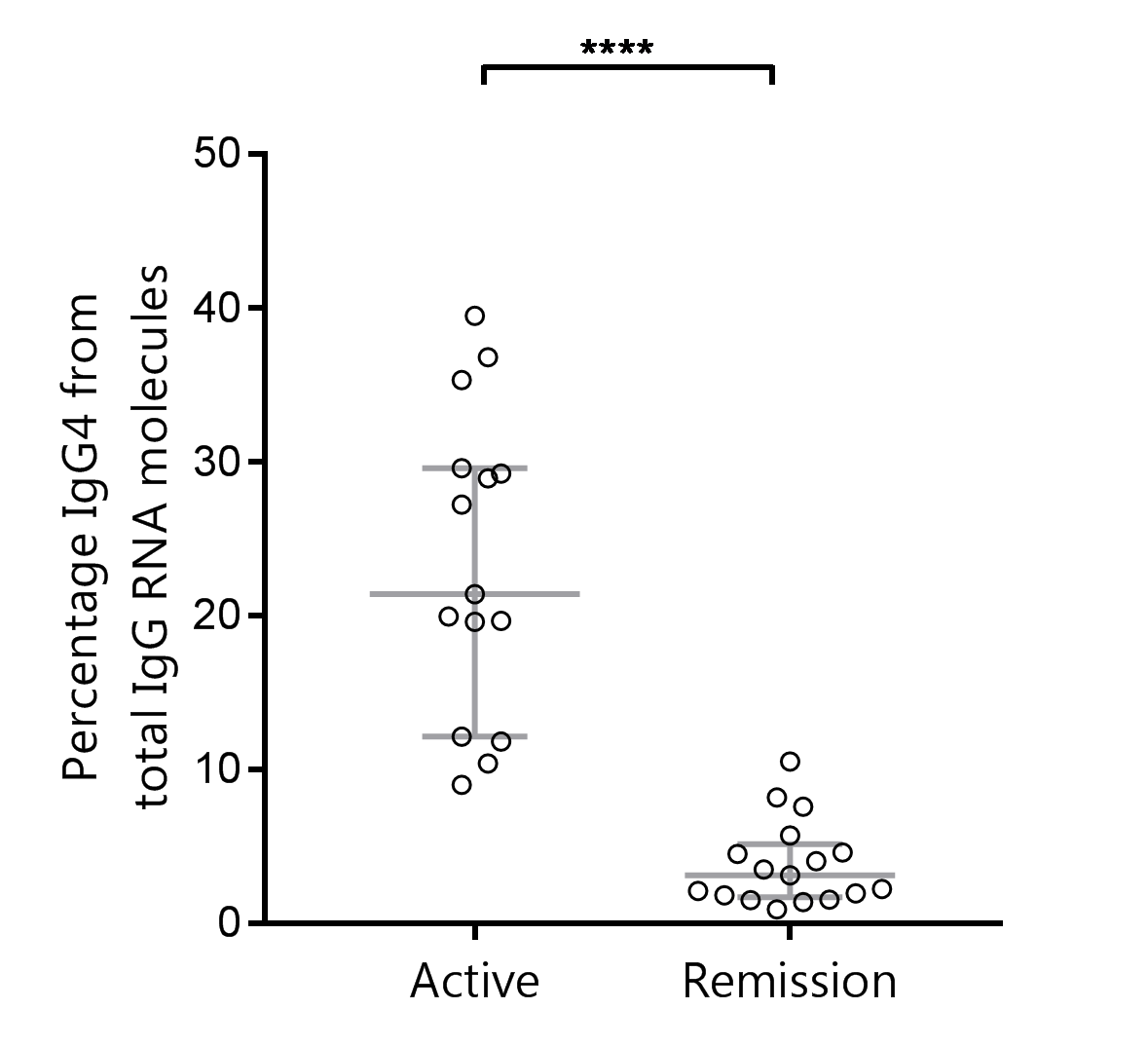

Supplement: Supplementary file 3 — Figure S2. Active GPA vs remission GPA (without LDA). Scatter dot plot portraying the percentage of IgG4 from total IgG RNA molecules in the active vs remission groups in GPA without LDA. (BMP 3716 kb) [file 13075_2018_1806_MOESM3_ESM.bmp]

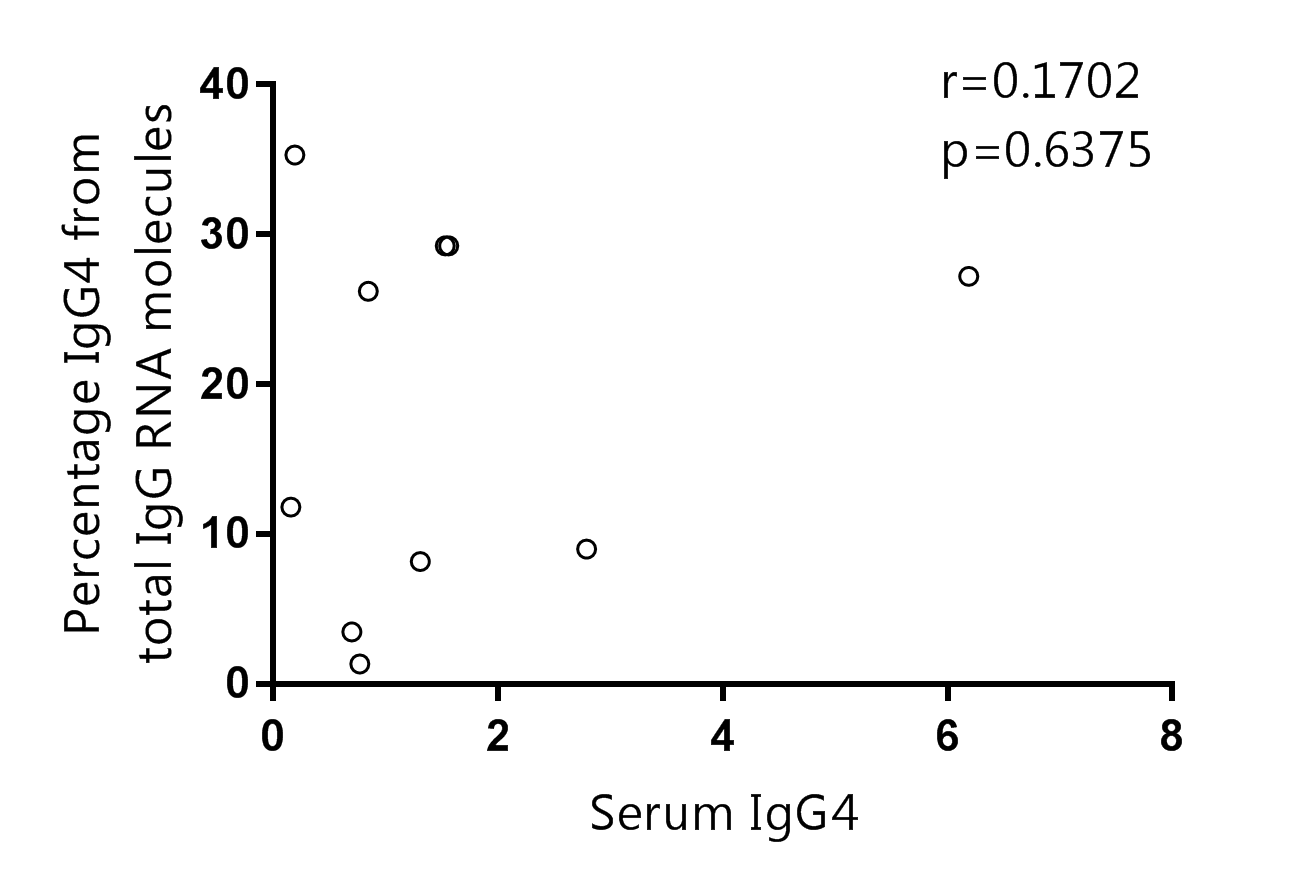

Supplement: Supplementary file 4 — Figure S3. Serum IgG4 vs qPCR test. Scatter dot plot portraying the correlation between serum IgG4 (x-axis) and the percentage of IgG4 from total IgG RNA molecules (y-axis) (Spearman’s r correlation). (BMP 3413 kb) [file 13075_2018_1806_MOESM4_ESM.bmp]

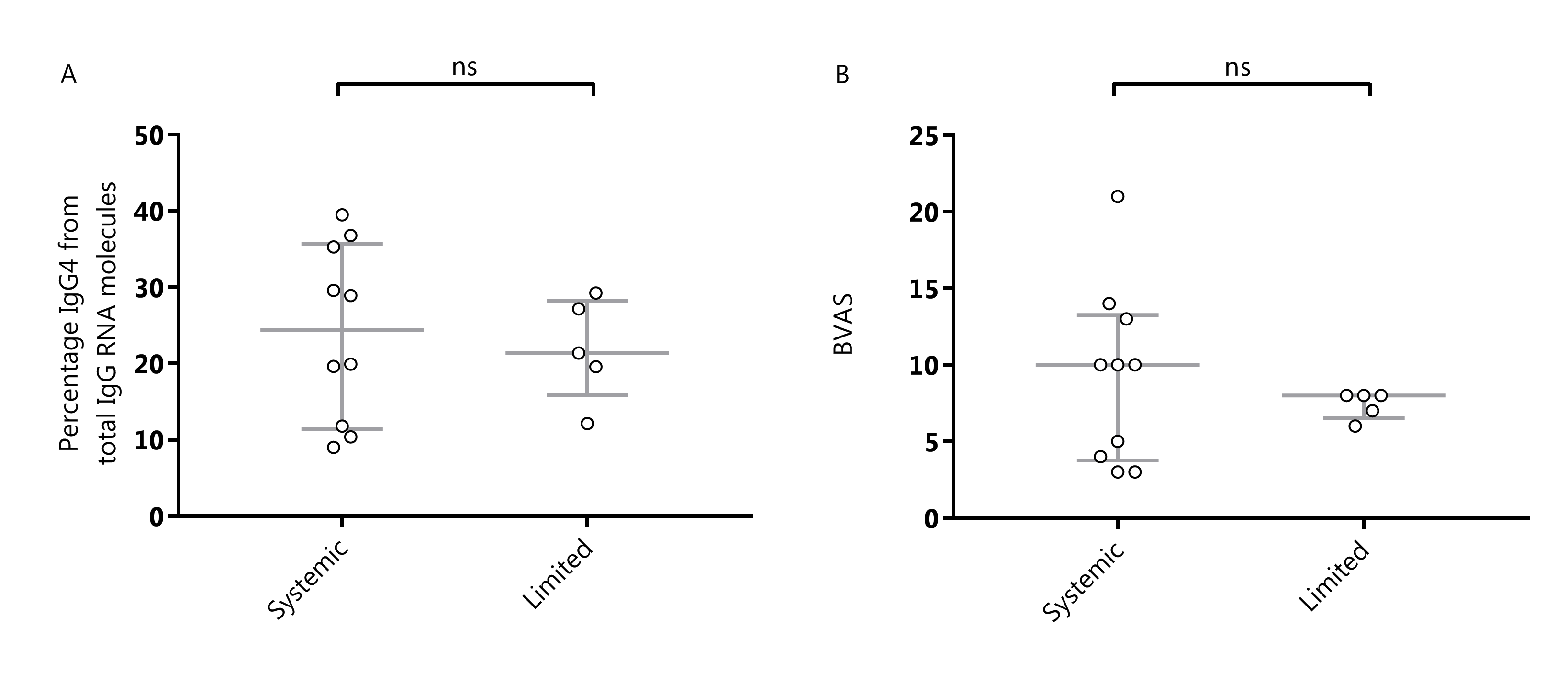

Supplement: Supplementary file 5 — Figure S4. Systemic GPA vs limited GPA. Scatter dot plot with in (a) the qPCR result within the active GPA group divided by systemic and limited disease and in (b) the matching BVAS for this group. (BMP 13494 kb) [file 13075_2018_1806_MOESM5_ESM.bmp]

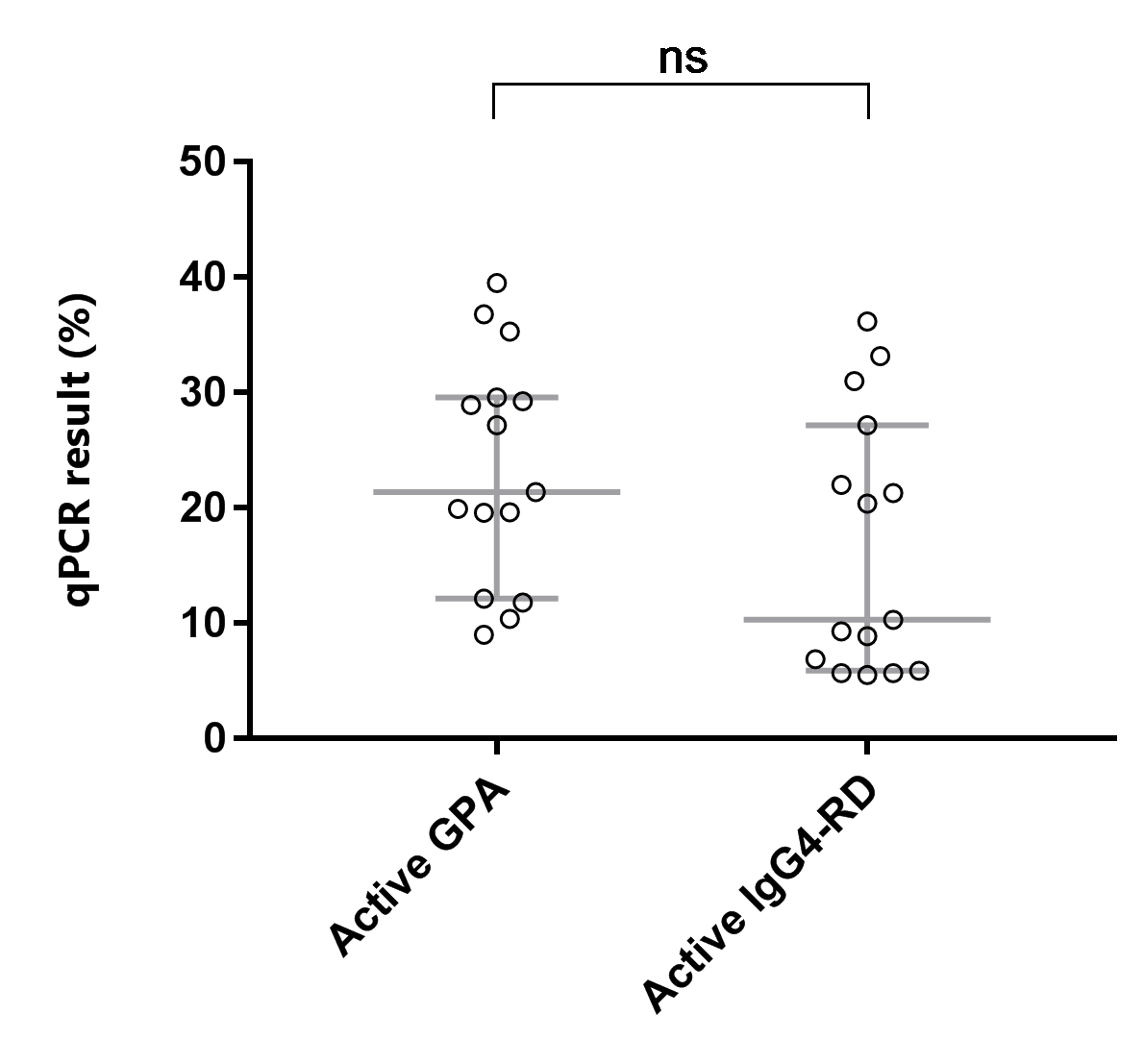

Supplement: Supplementary file 6 — Figure S5. Active GPA vs active IgG4-RD. Scatter dot plot portraying the percentage of IgG4 from total IgG RNA molecules in the active GPA vs active IgG4-RD control groups. ns = not significant. (BMP 3861 kb) [file 13075_2018_1806_MOESM6_ESM.bmp]

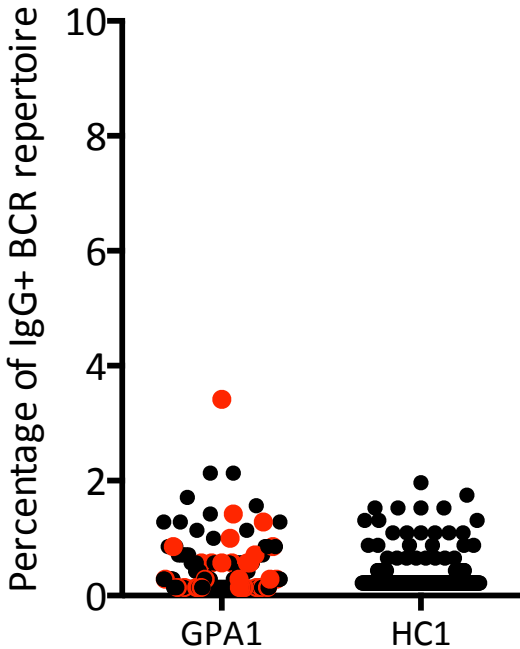

Supplement: Supplementary file 7 — Figure S6. NGS data on IgG4+ BCR clones. NGS data portraying the frequency of IgG4+ BCR clones (red) and IgG clones (black) in a GPA patient (x-axis, left) and a representative healthy control subject (x-axis, right). See an earlier study for a complete description of the amplification protocol [43]. Briefly, a linear amplification is performed using six primers specific for the V segment of the BCR heavy chain. Each primer has a nonbinding common sequence on the 5′ end for the second amplification step. The primers cover all known V-segment alleles. After purification, amplification is performed using the common sequence introduced by the V primers and a common primer on the boundary of the J segment and C segment. Primers are available upon request. Further processing was performed according to the protocol for the MiSeq platform (Illumina, San Diego, CA, USA), which was used for NGS. NGS results were analyzed using a custom pipeline [44]. (PDF 60 kb) [file 13075_2018_1806_MOESM7_ESM.pdf]

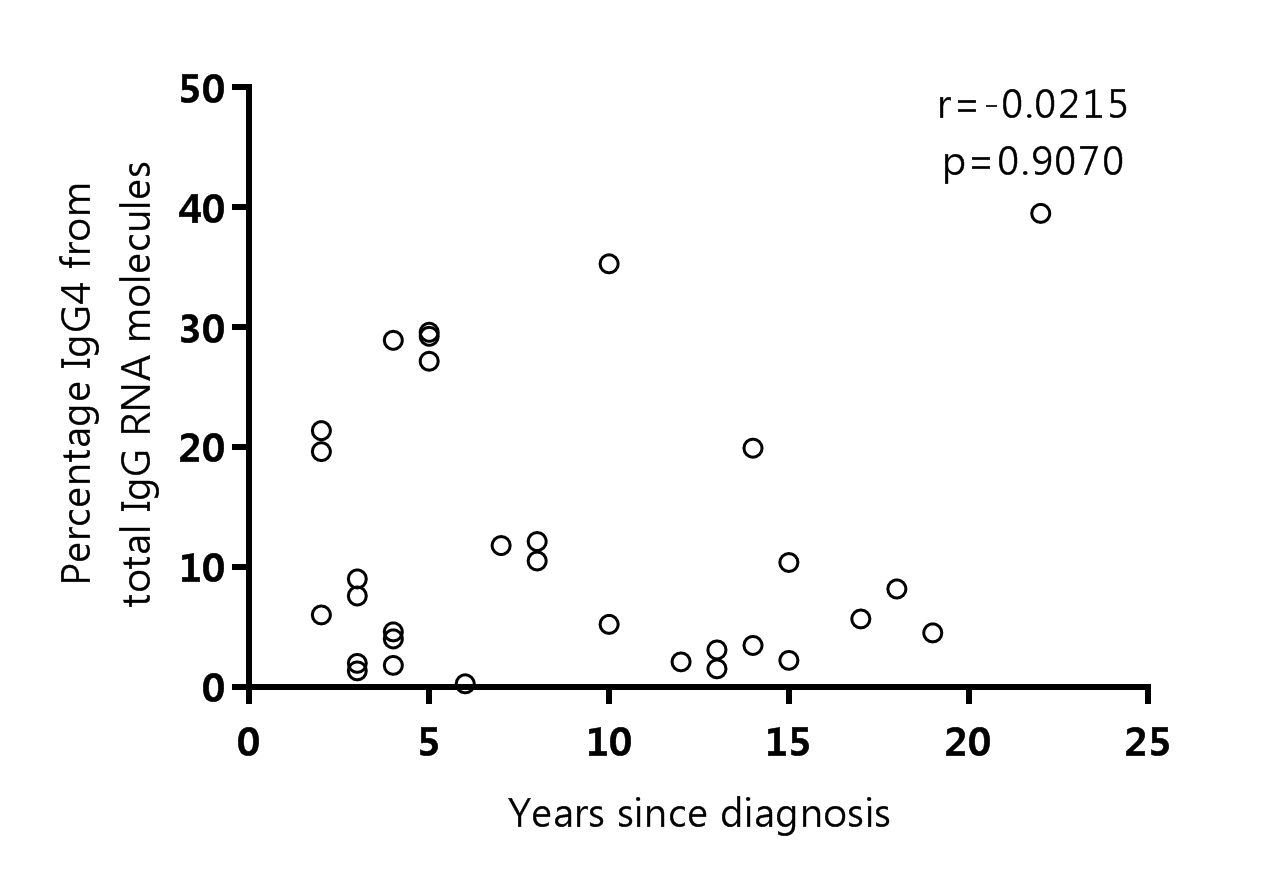

Supplement: Supplementary file 8 — Figure S7. Years since diagnosis vs qPCR score. The r and p values demonstrate no correlation between disease duration and qPCR score. (BMP 3328 kb) [file 13075_2018_1806_MOESM8_ESM.bmp]
